# Analyzing the gonadal transcriptome of the frog *Hoplobatrachus rugulosus* to identify genes involved in sex development

**DOI:** 10.1186/s12864-021-07879-6

**Published:** 2021-07-19

**Authors:** Yun Tang, Jing-Yi Chen, Guo-Hua Ding, Zhi-Hua Lin

**Affiliations:** 1 Laboratory of Amphibian Diversity Investigation, College of Ecology, Lishui University, Lishui, 323000 Zhejiang, People’s Republic of China; 2 College of Life Sciences, Nanjing Normal University, Nanjing, 210046 Jiangsu People’s Republic of China

**Keywords:** Assembly, Gene, Gonadal development, *Hoplobatrachus rugulosus*, Molecular pathway, Transcriptome

## Abstract

**Background:**

The tiger frog (*Hoplobatrachus rugulosus*) is listed as a national Class II protected species in China. In the context of global warming, the sex ratio of amphibians will be affected, and the development of the population will be limited. Therefore, considering the potential for a decrease in the number of amphibians, studying sex evolution and molecular regulation of gonadal development in *H. rugulosus*, phenomenon that are currently unclear, is of great significance.

**Results:**

Here, *H. rugulosus* was used to explore the mechanisms regulating gonadal development in amphibians. Illumina HiSeq 3000 was used to sequence the gonadal transcriptome of male and female *H. rugulosus* at two growth stages to identify genes related to gonadal development and analyze expression differences in the gonads. This analysis indicated that *cyp17α*, *hsd3β*, *hsd11β1*, *cyp19α*, and *hsd17β12* perform vital functions in sex development in amphibians. Specifically, the expression of *cyp3α*, *cyp17α*, *hsd3β*, *hsd11β1*, *sox2*, *sox9*, *sox30*, *soat*, *cyp19α*, *hsd17β12*, and *hspα1s* was correlated with gonadal development and differentiation in *H. rugulosus*, as determined using the quantitative reverse transcriptase-polymerase chain reaction.

**Conclusion:**

Significant differences were found in the gonadal gene expression levels in *H. rugulosus* of both sexes, and we identified a steroid hormone synthesis pathway in this species and analyzed related gene expression, but the changes during sex differentiation were still unclear. To our knowledge, this report presents the first analysis of the *H. rugulosus* gonadal transcriptome and lays the foundation for future research.

**Supplementary Information:**

The online version contains supplementary material available at 10.1186/s12864-021-07879-6.

## Introduction

Sex development in amphibians is a complicated process. As species that undergo genetic sex determination, the genotypic sex of amphibians is determined at the fertilization stage, but the phenotypic sex is influenced by the environment [1–3] and ultimately depends on the results of gonadal development. Therefore, the gonads represent the most appropriate organ for studying the sex of amphibians. Gonadal development in amphibians begins with the migration of primordial germ cells (PGCs) to the genital ridges, and then the PGCs and genital ridges co-develop into the primitive gonads, which further differentiate into testes or ovaries [4–6]. In most studies, the period from tadpole growth to the end of metamorphosis describes gonadal development [7–9], although a few studies have explored the complete progression from gonadogenesis to differentiation of the testes or ovaries and then to sexual maturation [10–12]. For example, Mali et al. [12] completely described the events that occurred at different stages of gonadal development in *Microhyla ornata* and *Hylarana malabarica* frogs and compared differences in their gonadal development during growth. Thus, previous research has established a relatively deep understanding of the processes involved in gonadal development in amphibians.

Modern biotechnology has made it possible to explore the molecular mechanisms underlying sex determination in amphibians. Many researchers have focused on genotypic sex determination systems and sex chromosomes of amphibians [13–16]. Some of the genes involved in sex development in amphibians, such as *dmrt1* [17–19], *sox3* [20], *sox9* [21, 22], *dax1* [23], *sf1* [24], *mis* [25], and *amh* [19], were discovered over the course of a few years. Researchers have identified the relevant genes through molecular-biology–based approaches and gained a preliminary understanding of their functions and mechanisms of action [19, 20]. Further, the only sex-determining gene *dm-w* has been found in *Xenopus laevis* [26]. Some reports have also shown the differential expression of some sex-related genes during gonadal development in amphibians [17, 27, 28]. However, the overall differences in the related gene expression levels during gonadal development in amphibians remain unknown.

During primary gonadal differentiation in amphibians, steroid hormones secreted by the gonads can control the development of accessory structures, and by extension, secondary sexual characteristics, which ultimately affects the sex phenotype [29]. Because of the special sex development in amphibians, the effects of steroid hormones on sex differentiation have also received extensive attention. Early researchers treated amphibians with exogenous steroid hormones and found that the phenotypic sex was reversed [30–32], which demonstrated the importance of steroid hormones in phenotypic sex differentiation in amphibians. In addition, previous findings have also indicated that the effects of the same steroid hormone are not uniform in different species; even in the same species, the effects can vary with different doses [14, 33, 34]. Several studies have also revealed that steroid hormones play important roles in early gonadal development [14, 15, 33, 35]. However, because the molecular mechanism of action is unclear, the functions of steroid hormones during gonadal development are controversial [14, 35]. Previous studies showed that genes associated with steroid hormone synthesis, such as *cyp11a1*, *star*, *hsd3b*, *cyp17*, *hsd17b*, and *cyp19* are expressed before gonadal differentiation [36]. Differential expression of *cyp19* has been reported in *Pleurodeles waltl* tadpoles of different genotypic sexes when the original gonads would soon develop into testis or ovaries [37]; this demonstrated the regulatory role of steroid hormones in gonadal differentiation. However, relevant research is still limited, and the differential expression of these steroid-hormone-synthesis–related genes during the entire gonadal development needs to be further studied.

Substantial research has been conducted on the molecular mechanisms underlying sex development in amphibians, although sex-related gene expression changes occurring during gonadal development are unclear, as are the regulation of sexual steroid hormones in amphibians and associated molecular pathways during gonadal development. Therefore, many problems remain to be solved, and further studies are needed. The main obstacle to resolving these questions is the lack of genetic and genomic information for amphibian species, although this lack of information can now be overcome using next-generation high-throughput sequencing technologies [38, 39].

*Hoplobatrachus rugulosus* (Wiegmann, 1834) is a member of Dicroglossidae within the Anura order in the Amphibia class. This species is listed in Appendix II of CITES as a national Class II protected species in China and represents a common frog in farmlands in southern China [40]. To strengthen the protection of this species, many scholars have begun studying the food habits, acoustic characteristics, growth, development, and physiology of this species [41–46]. Currently, the increase in global temperature owing to the greenhouse effect is modifying the sex of amphibians [14]. Thus, in the context of climatic warming, the sex ratio of amphibians will be affected and the development of this population will be limited. Therefore, considering the potential for a decreasing number of amphibians, based on the particularities of amphibian sex determination, studying sex evolution and the molecular regulation of gonadal development in *H. rugulosus* is of utmost significance. In this study, we generated millions of sequence reads from the gonadal transcriptome of male and female *H. rugulosus* at two growth stages, for the first time, using the Illumina HiSeq 3000 platform. A non-redundant set of transcripts was generated, various analyses were performed, and the expression of sex-related– and steroid-hormone-synthesis–related genes was further examined to evaluate the expression differences in gonadal glands. The results of this study provide a molecular foundation for future research.

## Results

### Anatomical observations and histological analysis of testes and ovaries

**Fig. 1:**
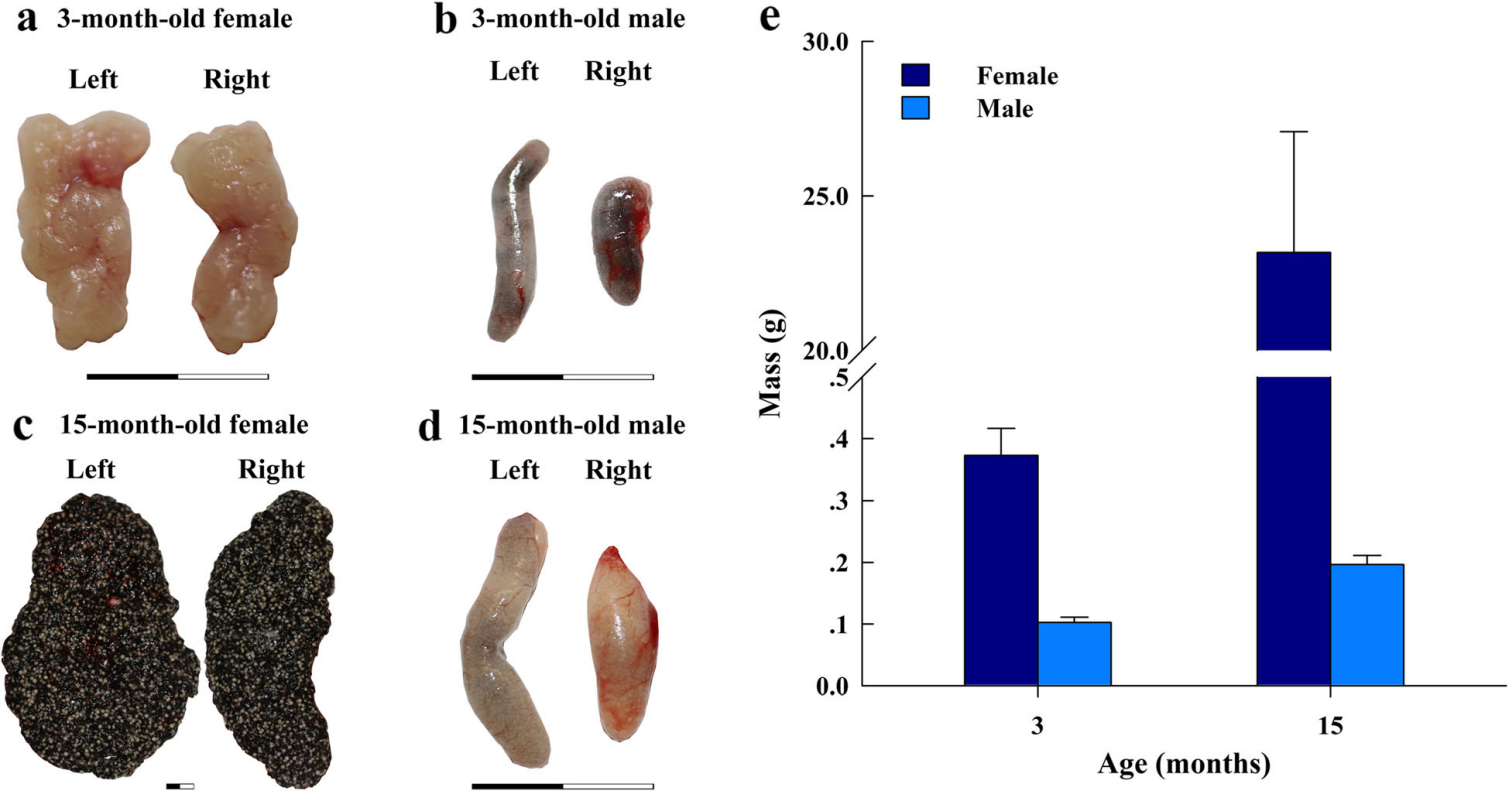
Anatomic observations of gonads from *Hoplobatrachus rugulosus* and comparison of their masses at two ages. **a** Left and right gonads from a 3-month-old female. **b** Left and right gonads from a 3-month-old male. **c** Left and right ovaries from a 15-month-old female. **d** Left and right gonads from a 15-month-old male. **e** Comparisons of the masses of gonads from males and females at two developmental stages. The scale bar represents 1 cm in the figure

**Fig. 2:**
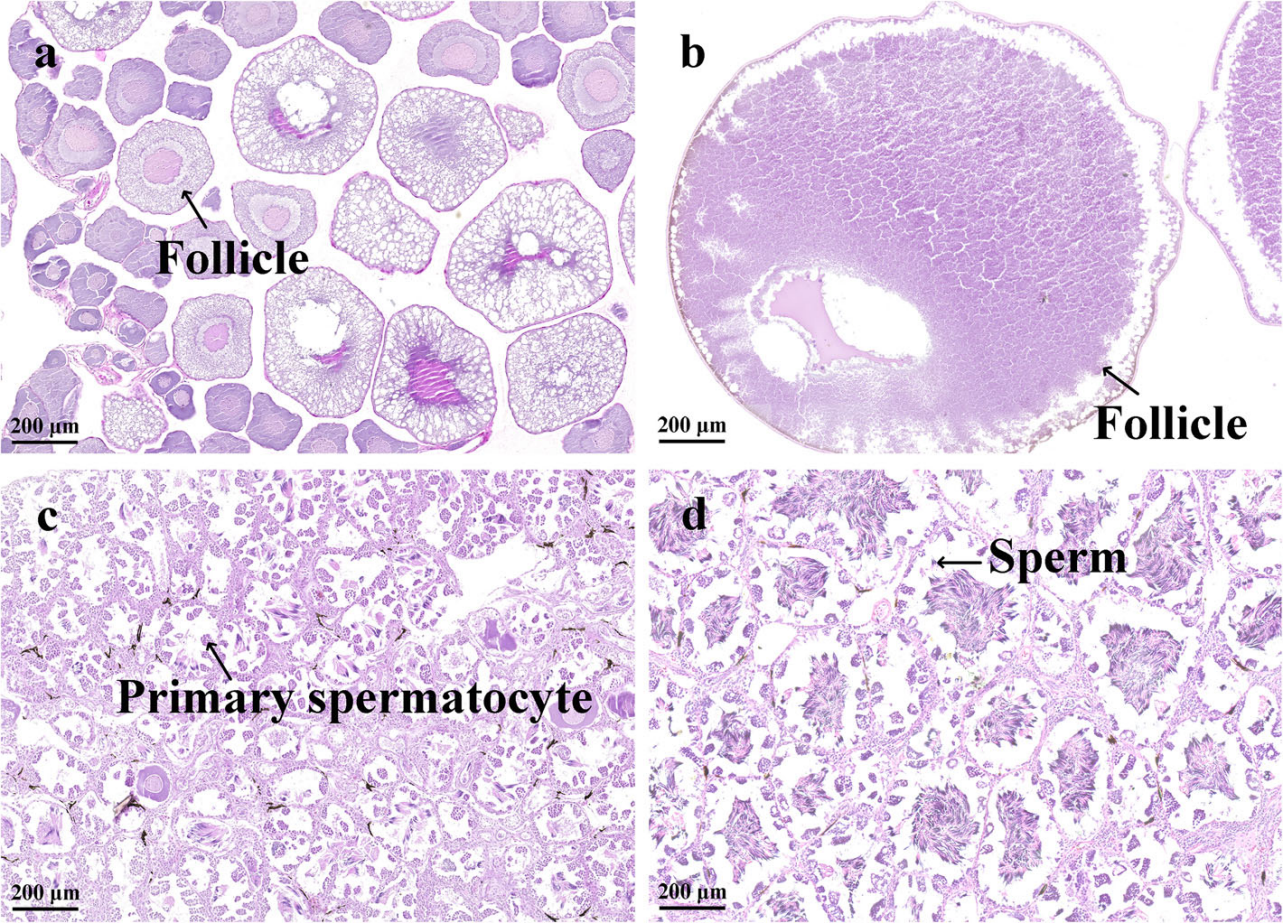
Histological observations of gonads from both sexes of *Hoplobatrachus rugulosus* at two ages. **a** Ovary from a 3-month-old female. **b** Ovary from a 15-month-old female. **c** Testis from a 3-month-old male. (d) Testis from a 15-month-old male

Male and female frogs that were reared for 3 months after complete metamorphosis exhibited differences in body structure and size; the vocal sac structures were visible in the males, but the gonads were immature (Fig. 1a, b). After 15 months, the males and females exhibited evident sexual dimorphism, the frogs had grown to become breeding individuals, and the gonads were fully mature (Fig. 1c, d); therefore, male and female gonad samples were obtained from 3- and 15-month-old *H. rugulosus* by dissection, and they were then observed and weighed. The ovaries exhibited apparent changes in size and morphology as the development progressed, age increased, volumes of the ovaries increased, and the color changed from pinkish white to black. In contrast, only the size of the testes changed, with little differences in the morphological characteristics. The mass of the gonads in 3-month–old females (hereafter referred to as 3F) was larger than that in 3-month–old males (hereafter 3 M; one-way ANOVA; F_1, 4_ = 37.0, *P* < 0.01), and the gonad mass increased with the age of *H. rugulosus* (Fig. 1e). The mass of the gonads in 15-month–old females (hereafter 15F) was greater than that in 15-month–old males (hereafter 15 M; one-way ANOVA; F_1, 4_ = 34.4, *P* < 0.01). We also prepared sections for histological analysis, which revealed the presence of growing follicles in the 3F gonads under a microscope. At 15 months of age, the follicles became larger and tended to mature. However, primary spermatocytes appeared in the 3 M gonads, and sperm cells were already present. Numerous sperm cells were already present in the 15 M gonads, and sexual maturation was achieved (Fig. 2).

### Sequence analysis and assembly

cDNA libraries were constructed using testes and ovaries to study the transcriptome of *H. rugulosus* at two ages. The analysis began with preprocessing and ended with comparisons of differentially expressed genes (DEGs) and functional annotations between different samples. After strict quality assessments and data filtering, 91,416,254 (96.53%) and 69,155,150 (95.07%) high-quality reads were generated from the 3 M and 3F groups, and 80,734,092 (91.31%) and 81,576,146 (91.23%) high-quality reads were generated from the 15 M and 15F groups, respectively, and these reads were selected for further analysis. The GC percentage of the reads in the 3 M group was 45.92%, that in the 3F group was 47.92%, that in the 15 M group was 45.92%, and that in the 15F group was 46.19% (Table S1). In addition, the respective Q20 and Q30 values of the DNA bases in the 3 M group after filtration were 94.36 and 86.51%, respectively, whereas those in the 3F group were 93.18 and 84.64%, those in the 15 M group were 91.89 and 82.48%, and those in the 15F group were 91.84 and 82.31% (Table S2). Therefore, the overall sequencing quality was good, and the data met the requirements for subsequent analysis. The Trinity program [47] was used to splice short RNA-seq sequences to obtain the reference genes, and 49,149 non-redundant unigenes were obtained. The average length obtained was 1593 base pairs (bp) and the N50 length was 2455 bp, which was indicative of a good-quality assembly (Table S3). The number of genes expressed in each sample was counted. Through this analysis, 48,910 unigenes were obtained from the 3 M samples, accounting for 99.51% of the total, 42,502 (86.48%) unigenes were obtained from the 3F samples, 48,588 (98.86%) unigenes were obtained from the 15 M samples, and 45,289 (92.15%) unigenes were obtained from the 15F samples (Table S4).

### Functional annotation and expression analysis

The obtained unigenes were analyzed using Nr, Swiss-Prot, Kyoto Encyclopedia of Genes and Genomes (KEGG), Clusters of Orthologous Groups (COG), and EuKaryotic Orthologous Groups (KOG) databases for functional annotation, and 20,942 unigenes were matched in at least one database. Among them, the most unigenes (20,925) matched with the Nr database, and the fewest unigenes (11,982) matched with the KOG database (Table S5). The annotation information is represented in a Venn diagram in Fig. S1. The E values of the best comparisons for all unigenes in the four major databases were counted and divided into five ranges (10–20 < E value ≤10–5; 10–50 < E value ≤10–20; 10–100 < E value ≤10–50; 10–150 < E value ≤10–100; 0 ≤ E value ≤10–150), and the number of genes in each range was counted. Nr is a well-known protein database, which enables the use of BLASTx to compare assembled unigene sequences with the Nr database to obtain the sequence with the best alignment result (lowest E value) of each unigene in the Nr database as the corresponding homologous sequence. In cases of equal E values, the first value is used. The number of homologous sequences was statistically compared to each species to identify the species to which the homologous sequences belong, and the top three species were all amphibians (Fig. S2). The homologous sequences with the highest similarity were from *Nanorana parkeri*, belonging to the same family as *H. rugulosus*, which validated our comparisons. In the KOG database (Fig. S3), 11,982 unigenes were grouped into 25 categories, and “signal transduction mechanisms” accounted for the highest proportion (5209, 43.47%), followed closely by “general function prediction only” (3814, 31.83%). ln contrast, “nuclear structure” (80, 0.67%) and “cell motility” (52, 0.43%) accounted for the lowest proportions, which suggested that most of the gonadal functional genes of *H. rugulosus* were used for signal transduction and basic cellular activities.

Next, we calculated and analyzed the gene expression levels in each sample, and generated a bar chart (Fig. S4A) showing the statistical correlations of DEGs among the samples. Pairwise comparisons of DEGs were performed using the four samples, revealing the following results. Comparison of the 3F and 3 M groups revealed the presence of 29,166 upregulated genes and 8579 downregulated genes in the 3 M group (Table S6). Comparison of the 15F and 15 M groups revealed the presence of 24,660 upregulated genes and 10,879 downregulated genes in the 15 M group (Table S7). Comparison of the 3F and 15F groups revealed the presence of 12,786 upregulated genes and 3074 downregulated genes in the 15F group (Table S8). Finally, comparison of the 3 M and 15 M groups revealed the presence of 3357 upregulated genes and 5847 downregulated genes in the 15 M group (Table S9). These differences might reflect the fact that the 15 M and 3 M samples were derived from testes, whereas the 15F and 3F samples were from ovaries, which represent different tissue types, thus resulting in increased numbers of DEGs. In addition, genes expressed in the testes were more upregulated and less downregulated than those expressed in the ovaries at both 3 and 15 months of age. Comparison of the ovary samples at 3 and 15 months of age indicated that more genes were upregulated and fewer genes were downregulated at 15 months, whereas the opposite results were obtained when analyzing the testes samples. The scatter plot in Fig. S4B reveals greater correlations between samples obtained from females (3F and 15F) and males (3 M and 15 M), compared with samples obtained from different sexes. The volcanic plots of the four samples more intuitively show the expression levels of DEGs in gonads from different sexes at two developmental stages (Fig. S4C).

### Enrichment analysis of DEGs

**Fig. 3:**
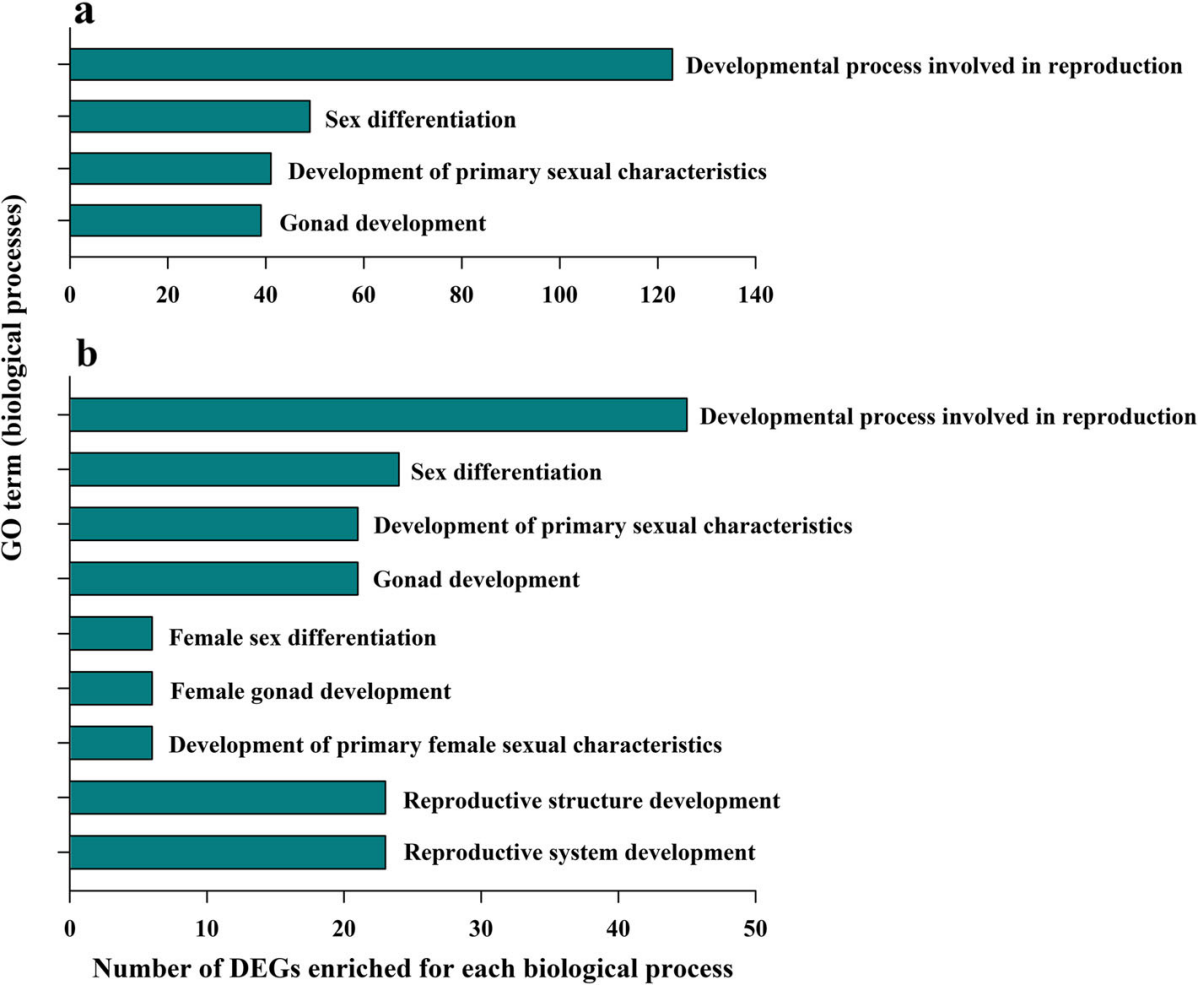
Gene Ontology (GO) term enrichment analysis. **a**, **b** GO terms (related to biological processes) significantly enriched (*P* < 0.05) for the differentially expressed genes (DEGs) identified when comparing the 3F group with the 3 M group (**a**) or the 3F group with the 15F group (**b**). 3F: 3-month-old female, 3 M: 3-month-old male, 15F: 15-month-old female, 15 M: 15-month-old male

We carried out Gene Ontology (GO)/KEGG pathway enrichment analysis of the DEGs between the different groups, and GO functions and KEGG pathways associated with the DEGs were determined. GO analysis is mainly divided into three categories, molecular function, cellular components, and biological processes. Through GO functional-enrichment analysis, we classified DEGs obtained by pairwise comparisons, and selected GO terms (and the corresponding GO numbers) that might be related to gonadal development. Comparison of the 3F group with the 3 M group revealed 23 GO terms related to gonadal development or function, among which the DEGs were significantly enriched in biological processes such as gonadal development (GO:0008406), development of primary sexual characteristics (GO:0045137), sex differentiation (GO:0007548), and developmental process involved in reproduction (GO:0003006), among others (Fig. 3). Comparison of the 15F group with the 15 M group revealed 25 GO terms related to gonadal development or function, although no DEGs were significantly enriched. Comparison of the 3F group with the 15F group revealed 20 GO terms related to gonadal development or function, with significant enrichments in biological processes like the development of primary sexual characteristics (GO:0045137), sex differentiation (GO:0007548), gonad development (GO:0008406), development of primary female sexual characteristics (GO:0046545), female gonad development (GO:0008585), female sex differentiation (GO:0046660), developmental process involved in reproduction (GO:0003006), reproductive structure development (GO:0048608), and reproductive system development (GO:0061458). Finally, upon comparing the 3 M group with the 15 M group, we found that 17 GO terms were related to gonadal development or function, but no significant enrichment was found in terms of the DEGs. Through significant pathway enrichment, the major biochemical metabolic pathways and signal transduction pathways in which DEGs are involved can be determined.

To determine active biological pathways during the sexual development of *H. rugulosus*, we carried out pathway enrichment analysis of the DEGs. Comparison of the 3F group with the 3 M group revealed that the DEGs were enriched in 340 KEGG pathways. Comparison of the 15F group with the 15 M group revealed DEGs that were enriched in 339 KEGG pathways. Comparison of the 3F with the 15F group revealed DEGs that were enriched in 334 KEGG pathways. Finally, comparison of the 3 M group with the 15 M group revealed DEGs that were enriched in 321 KEGG pathways. Some of the signaling pathways identified are essential for development, including steroid hormone biosynthesis (ko00140), ovarian steroidogenesis (ko04913), Gnrh signaling pathway (ko04912), cortisol synthesis and secretion (ko04927), estrogen signaling pathway (ko04915), steroid biosynthesis (ko00100), oocyte meiosis (ko04114), and progesterone-mediated oocyte maturation (ko04914). Further information regarding these predictive pathways might be useful for investigating the roles played by these DEGs during the gonadal development of *H. rugulosus*.

### Quantitative reverse transcriptase PCR (qRT-PCR)

**Fig. 4:**
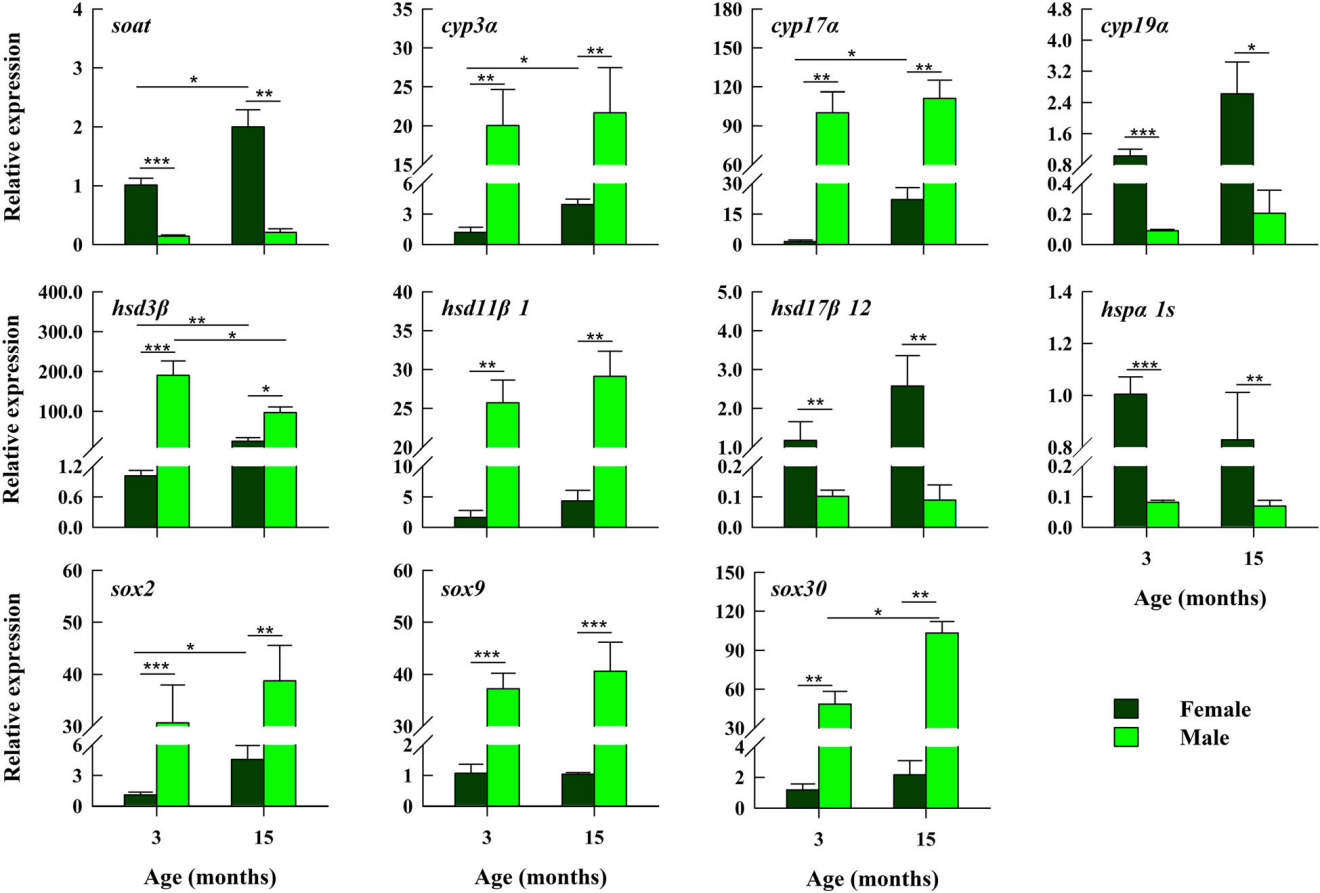
qRT-PCR analysis of 17 differentially expressed genes (DEGs) in male/female gonads from *Hoplobatrachus rugulosus* at two ages. *Gapdh* expression was detected as a reference gene to normalize the qRT-PCR data. Data are expressed as the mean + standard error. *P < 0.05; **P < 0.01; ***P < 0.001

**Fig. 5:**
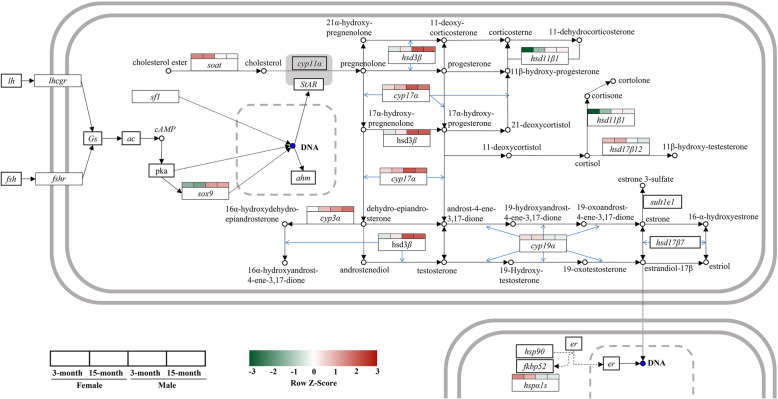
Differential expression of sex-related genes in male/female gonads from *Hoplobatrachus rugulosus* at two ages. The original pathways (ko00140, ko04913, ko04927, ko04915, ko00100, and ko04024) from KEGG database (https://www.kegg.jp/kegg/; Permission: 210365)

After analyzing the overall transcriptome data, we filtered out the important DEGs associated with sex development and steroid hormone synthesis, and these results were verified by qRT-PCR. We compared the qRT-PCR results for 11 genes related to sex development with the transcriptomics results (Fig. 4). *Cyp3α*, *cyp17α*, *hsd3β*, *hsd11β1*, *sox2*, *sox9*, and *sox30* showed higher expression levels in males than in females, whereas *soat*, *cyp19α*, *hsd17β12*, and *hspa1s* showed the opposite results (one-way ANOVA, all *P* < 0.05). *soat*, *cyp3α*, *cyp17α*, and *sox2* showed higher expression levels in females at 15 months of age than at 3 months of age, whereas the *sox30* gene showed the opposite result, and *hsd3β* gene expression was lower in the 15 M group than that in the 3 M group (one-way ANOVA, all *P* < 0.05; Fig. 4). In addition, most KEGG pathway-related genes were related to steroid hormone synthesis. After sorting and simplifying these pathways, we obtained the pathway map shown in Fig. 5. We used coloring to mark the sex-related genes in the pathway map for different groups to present the reads per kilobase per million mapped reads (RPKM) data more intuitively.

## Discussion

High-throughput transcriptome sequencing is widely used in various molecular studies [48]. Compared to traditional sequencing technology, high-throughput sequencing technology using the Illumina platform offers unparalleled advantages such as high throughput, high accuracy, and low cost; thus, the platform has become a powerful tool for full transcriptome research. Our study represents the first attempt to study the gonadal transcriptome of *H. rugulosus*. In total, 49,149 unigenes with an average length of 1593 bp were obtained, and the transcriptomes of the gonadal glands at different periods in different sexes were compared. The results are of great significance for the further study of gonadal development and sex differentiation in *H. rugulosus*.

In this study, the four groups were compared in pairs. We identified far more DEGs when comparing both sexes than when comparing both ages. These results might have been obtained because although both the testis and the ovary develop from the same original gonads, the testes and ovaries (two different sex organs) form owing to the differential expression of sex-related genes during gonadal differentiation [19]. Previously, several sex-related genes were found in amphibians [19–22, 24], and the expression levels of these were different in the testes and ovaries. For example, the *amh* gene of the TGF family [49, 50] is expressed in differentiated testicular Sertoli cells, but it is also expressed in the ovaries. Therefore, it was proposed that its function is not limited to testicular development [19]. The *dmrt1* gene is involved in male sex development [51, 52] and is highly expressed in male germ cells as well as somatic cells during amphibian development, but it is also expressed at low levels in some amphibian ovaries [19]. The *sox9* gene, a member of the SRY-related HMG-box (*sox*) family, is expressed in both the testes and ovaries, but the expression level in the testes is higher than that in the ovaries [22]. The results of these studies identified significant differences in gonadal gene expression among amphibians of different sexes, consistent with our current results with *H. rugulosus*.

In addition, more DEGs were upregulated and fewer were downregulated in the testes than those in the ovaries, at both stages of development, in previous reports for other species. Similar results were found in studies of gene differences in the testes and ovaries of *Portunus trituberculatus* [53] and *Oratosquilla oratoria* [54], suggesting that more genes were highly expressed in the testes than in the ovaries and that they were involved in testicular development or the regulation of biological processes. However, few studies on this topic have been conducted in amphibians to date, and the gene expression levels in gonads from different sexes remain to be further explored. When comparing pairs of two ages, more genes were upregulated and fewer were downregulated in the 15F ovaries than in the 3F ovaries, whereas the opposite was true in the testes at two developmental stages. Therefore, we suspected that during development, as the ovaries of females progressed from an immature to mature stage, more genes were involved in ovarian development. However, in the testes, more genes were actively expressed during the early stage of development, and these genes were downregulated during the progression from immature to mature stages. There is a lack of data regarding gonadal gene expression during development in amphibians; thus, our research provides a more in-depth understanding of this aspect of development and provides a reference for further study of the dynamic gene expression changes that occur during gonadal development in amphibians.

GO enrichment analysis of DEGs between the different groups showed that terms such as gonadal development, development of primary sexual characteristics, sex differentiation, and developmental process involved in reproduction were enriched for different genes between the 3F and 3 M groups and between the 3F and 15F groups. These results suggest that these DEGs played important roles as potential regulatory factors during the early gonadal development of females. In addition, by performing pathway enrichment analysis, we generated pathway maps for gonadal development-related pathways, such as steroid hormone biosynthesis, ovarian steroidogenesis, the Gnrh signaling pathway, cortisol synthesis and secretion, the estrogen-signaling pathway, steroid biosynthesis, oocyte meiosis, and progesterone-mediated oocyte maturation. Most of these pathways were related to the synthesis of gonadal steroid hormones, which confirmed the important role of steroid hormones in the development and differentiation of gonads in *H. rugulosus*, whereas the formation of gonads is largely dependent on steroid hormones [55]. It was also previously found that testosterone (T) and estradiol (E2) in amphibian tadpoles are naturally synthesized before gonad differentiation, and the gonads directionally differentiate after binding to the corresponding sex hormone receptors to form complexes [15, 16], which provides a foundation for investigating the molecular mechanisms regulating steroid hormone expression in amphibians. To more clearly analyze the molecular mechanisms underlying sex differentiation in *H. rugulosus*, we simplified the pathway, screened sex-related GO terms enriched in some important DEGs in the pathway and verified the results by qRT-PCR.

After qRT-PCR verification of the genes screened for sex development and steroid synthesis, the expression trends of 17 genes were consistent with the results obtained upon transcriptome analysis, among which 11 genes were selected for further analyses. The remaining six genes were expressed differently in other pathways instead of the steroid synthesis signaling pathway, and there was no significant difference in the expression of upstream and downstream genes; their specific role and function are unknown, and therefore, we did not analyze them. Among them, *cyp3α*, *cyp17α*, *hsd3β*, *hsd11β1*, *sox2*, *sox9*, and *sox30* showed higher expression levels in males than in females, whereas the opposite results were found for *soat*, *cyp19α*, *hsd17β12*, and *hspα1s*. Figure 5 shows that *cyp17α*, *hsd3β*, *hsd11β1*, *cyp19α*, and *hsd17β12* play important roles in the steroid hormone synthesis pathway, and the functions of *cyp17α* and *cyp19α* have been confirmed in numerous studies [6, 16, 55, 56]. It is known that cytochrome P450 17-hydroxylase/17, 20 lyase (*cyp17α*) can promote the conversion of progesterone into dehydroisoandrosterone and that cytochrome P450 aromatase (*cyp19α*) can transform T into E2 (Fig. 5). Indeed, *cyp17α* gene expression is upregulated in male tadpoles before sex determination and maintained a high expression level, and *cyp19α* is highly expressed in the undifferentiated gonads of female tadpoles [36]. Data from several studies have shown that increased expression of aromatase is a major indicator of ovarian differentiation in non-mammalian vertebrates, such as birds, reptiles, and various amphibians [22]. Therefore, the importance of the *cyp19α* gene in amphibian sex differentiation is evident.

Based on these data, in combination with our current results, we propose that during amphibian sex differentiation, transcription factors promote *cyp17α* expression in *H. rugulosus* at the early stage, some of which develop into males owing to low *cyp19α* expression and high *cyp17α* expression, whereas some have high gonadal *cyp19α* gene expression, which promotes the differentiation of ovaries and the development of females. Figure 5 shows that follicle-stimulating hormone (*fsh*) and luteinizing hormone (*lh*) regulate the production of corresponding hormones by binding to specific receptors (*fshr* and *lhcgr*, respectively), and resulting in the activation of protein kinase A and promotion of *sox9* expression. Previous findings have shown that *sox9* is associated with the development of male gonads [21, 57]. Our results showed that *sox9* expression was higher in male gonads than that in female gonads, which also demonstrated a correlation between *sox9* and male sex development. Other genes with significant differences have not been reported in the literature for amphibians. However, *sox2*, *sox30*, and *sox9* all belong to the *sox* family, and *sox30* gene expression has been related to gonadal development in other species [58]. Therefore, its function might be similar to that of *sox9*, and further experimental studies are needed on this topic. In reptiles, the sex of most species is related to temperature [59–61], and heat shock protein (*hsp*) genes have also been considered relevant candidate genes, owing to their temperature-sensitive expression [62]. *Hsp70* family genes are necessary for translocation and protein folding [63]. Previous results have shown that the *hsp70α* mRNA expression is significantly higher in ovaries than that in testes at the same age [62]. In this study, *hspα1s* (a member of the *hsp70* family) was highly expressed in female gonads, and previous data indicated that the sex differentiation process in amphibians is also affected by temperature [14]. These findings suggest the importance of *hsp70* in gonadal development in species for which sex is affected by temperature.

In addition, *soat*, *cyp3α*, *cyp17α*, *sox2*, and other genes showed higher expression in females at 15 months of age than at 3 months of age, whereas the opposite results were found for *sox30*. These findings indicate that *soat*, *cyp3α*, *cyp17α*, *sox2*, and other genes might play an important role in mature female gonads. As our study was conducted only at 3 and 15 months of age, we were unable to show a continuous change in development, and whether these genes play increasingly important roles during the continuous sex differentiation of females still needs to be tested. The *sox30* gene is thought to be related to gonadal development, spermatogonial differentiation, and spermatogenesis [58], supporting our observations that *sox30* did not play an obvious role in female gonads, and we hypothesized that its expression decreased as the female gonads matured. However, as our sample was only analyzed at two time points, and as we cannot infer the entire developmental process, and this speculation needs to be further tested. In addition, *hsd3b* expression was lower in males at 15 months of age than at 3 months of age. Some scholars have reported that the sex-related gene *sf1* is dimorphologically expressed in amphibians of different sexes but that its expression is upregulated in females and downregulated in males, with increasing age [24]. These data suggest that the genes studied here might have also underwent dimorphism changes between both sexes and that their expression levels changed according to different needs during ontogenesis. However, few reports have described changes in the expression levels of sex-related genes during development, which highlights a need for further study and verification in the future.

## Conclusions

Our experimental results supported the following conclusions. (1) Significant differences were found in the gonadal gene expression levels in *H. rugulosus* of both sexes, and compared with those in ovaries, more genes were highly expressed in the testes and were involved in testicular development or in regulating testes-related biological process. (2) The important roles played by steroid hormones in *H. rugulosus* gonad differentiation were confirmed, and the major regulatory pathways of steroids during sex development were integrated through enrichment with GO and KEGG analyses. Specifically, the crucial functions of *cyp17α*, *hsd3β*, *hsd11β1*, *cyp19α*, and *hsd17β12* were identified, although our results do not yet indicate which transcription factors promoted the expression of these genes. (3) qRT-PCR revealed that the expression of *cyp3α*, *cyp17α*, *hsd3β*, *hsd11β1*, *sox2*, *sox9*, *sox30*, *soat*, *cyp19α*, *hsd17β12*, and *hspα1s* differed between both sexes and ages, and therefore, we speculate that these genes are closely related to gonadal development and differentiation in *H. rugulosus*. However, our paper is limited by certain shortcomings. As we only focused on two time points in the developmental process, we could not observe the continuous changes in gene expression during gonadal development, and thus, during development, it is unknown if more genes are involved in ovarian development as the females mature gradually. Moreover, in the testes, more genes were upregulated during the early stage of development, but it is unclear if the expression of these genes decreased gradually with testicular development. Further verification is needed.

## Materials and methods

### Ethics statement

The experimental procedures used in this study complied with the current laws related to animal welfare and research in China and were specifically approved by the Animal Research Ethics Committee of Lishui University (permit number ARECLSU201606001).

### Sample collection and preparation

The female and male frogs used in this study were collected from our froggery at Lishui University, China. Three frogs of each sex were collected at 3 and 15 months of age, which covered two gonad development stages. In each group, the coefficient of variation of snout-vent length and the body mass of selected frogs ranged from 2.9 to 8.9% and from 3.5 to 12.7%, respectively, and the details are shown in the Table S10. After transfer to our laboratory, each frog was individually reared in a 500 × 360 × 200 mm plastic tank placed in a room for 1 week, where the temperature varied naturally, often within the range of 26 °C to 30 °C. After anaesthetization with 0.05% MS-222 (Sigma), the ovary and testis tissues were collected. Next, samples from frogs of the same sex and age were mixed together and transferred to 2 ml RNase-free plastic tubes. These frogs were derived from the same clutch of tadpoles, and they grew up in the same rearing environment, which could eliminate the differences within the same group caused by genetic and environmental backgrounds. Samples were immediately frozen in liquid nitrogen and stored at − 80 °C.

### RNA extraction, library construction, and sequencing

Ovary (or testis) tissues from frogs at each age were subjected to RNA extraction. Total RNA was extracted using the Trizol Reagent Kit (Sangon Biotech, Shanghai, China) following the manufacturer’s protocol. RNA quality (RIN values ranged from 7.5 to 8.7) was assessed using an Agilent 2100 Bioanalyzer (Agilent Technologies, Palo Alto, CA, USA) and confirmed using RNase-free agarose gel electrophoresis. Equal amounts of high-quality RNA from each ovary or testis sample (three samples were mixed for per age group) were then pooled for RNA-seq analysis. Oligo (dT) beads were used to purify mRNA from extracted total RNA samples, and the mRNAs were fragmented into short fragments using fragmentation buffer (NEB #E7490, New England BioLabs). The fragmented mRNA was used as the template, and the first strand cDNA was synthesized by random hexamers; the second strand cDNA was then synthesized by adding the buffer, dNTPs, RNase H, and DNA polymerase I. After purification with a QIAquick PCR Purification Kit and elution in EB buffer, the obtained products were suspended into End Repair Mix for end reparation and adenylation of the 3′ ends. Following addition of the sequencing adaptors, fragments with the targeted size (200 bp) were recovered by agarose gel electrophoresis (A620014, Agarose, Sangon Biotech, Shanghai), after which PCR amplification was performed to create the final cDNA library. The PCR products were sequenced using the Illumina HiSeq 3000 sequencing platform. Preparation and sequencing of the cDNA library were implemented by Genedenovo Biotechnology Co., Ltd. (Guangzhou, China).

### De novo assembly of sequencing reads

The original imaging data produced were converted to sequence data using Base Calling software (bcl2fastq v2.20.0.422, Illumina), which we used to call raw data or raw reads, and cleanup was performed using Fastp software v0.18.0 [64]. In addition, the sequencing depth was 8G, paired-end sequencing was used, and the sequencing strategy was PE150. Not all clean reads obtained were valid, and reads containing adaptor, repetitive, or low-quality sequences could affect the assembly and subsequent analysis. Thus, we used Fastp to filter the clean reads by deleting adaptor sequences, reads where the proportion of Ns was > 10%, and low-quality reads (Q20 accounting for > 40% of the total reads). After recovering the filtered high-quality clean reads, the sequencing qualities were evaluated using Fastp. Subsequently, Trinity software v2.8.4 [47] was used for de novo transcriptome assembly. Reads with a 30 bp length of overlap were connected to form longer fragments with Trinity software (k-mer = 31), and these N-free assembled reads were assembled to generate unigenes.

### Functional annotation and DEGs

The basic functional annotations of unigenes included protein functional annotations, pathway annotations, and functional annotation with the COG and KOG databases (http://www.ncbi.nlm.nih.gov/COG). First, the unigene sequences were aligned to protein databases such as the Nr (date of access: 2020-05-14), SwissProt (date of access: 2020-05-14), KEGG (version number: Release 93.0), and COG/KOG (date of access: 2015-07-24) databases (e value < 0.00001) using BLASTx with default parameters (June, 2019), the protein with the highest sequence similarity to a given unigene was selected, and the protein functional annotation information of the unigene was obtained. Then, the RPKM method [65] was used to calculate the expression levels of unigenes for four samples, using the following formula: RPKM = (1,000,000 × C)/([N × L]/1000). To calculate the expression level of unigene A, C represents the number of reads for unigene A, N is the total number of reads that uniquely aligned to all genes, and L is the number of bases in unigene A. The RPKM method can be used to eliminate the influence of the gene length and sequence-quantity differences on the calculated gene expression level. The calculated gene expression level can be directly used to compare the gene expression differences in different samples, using the edgeR program (http://www.bioconductor.org/packages/release/bioc/html/edgeR.html) for statistical analysis. The false-discovery rate (FDR) and log_2_ fold-change (FC) were obtained for each unigene, and DEGs were identified using screening criteria of FDR < 0.05 and |log_2_ FC| > 1.

### GO enrichment analysis

We performed GO functional analysis of the DEGs identified among the samples [66]. The GO database employs three ontologies to describe the molecular functions, cellular components, and biological processes of genes, and GO analysis was performed using the DEGs from the edgeR analysis. The calculation was performed using Eq. 1, as follows:

$$ P=1-\sum \limits_{i=0}^{m-1}\frac{\left(\begin{array}{l}M\\ {}i\end{array}\right)\left(\begin{array}{l}N-M\\ {}n-i\end{array}\right)}{\left(\begin{array}{l}N\\ {}n\end{array}\right)} $$

where N is the number of genes with the GO annotation, n is the number of DEGs in N, M is the number of all genes that are annotated to certain GO terms, and m is the number of DEGs in M. Using an FDR of 0.05 as the threshold, the *P* value was calculated after the FDR was corrected, and then, a hypergeometric test was applied using the R v3.2.1 to identify the GO entries that were significantly enriched in DEGs, when compared with the whole genome background.

### KEGG pathway enrichment analysis

The Pathway database is the main component of the public KEGG database (https://www.kegg.jp/kegg/) [67]. In organisms, different genes can exert biological effects in a coordinated manner, and pathway-based analysis is helpful for further understanding the biological functions of genes. KEGG pathway analysis was used to analyze datasets to identify significantly enriched genes, and a hypergeometric test was used to identify pathways that were significantly enriched for DEGs compared with the whole genomic background. Eq. 1 was also used for KEGG enrichment analysis. Specifically, N is the number of genes with a KEGG annotation, n is the number of DEGs in N, M is the number of specific pathways associated with all gene annotations, and m is the number of DEGs in M. Using FDR = 0.05 as the threshold, the *P* value was calculated after the FDR was corrected. Pathways that met this condition were defined as those with significant enrichment for DEGs.

### qRT-PCR analysis

In this study, we also screened for DEGs important for sex development and steroid hormone synthesis and verified the expression levels of these genes by qRT-PCR [68]. For the qRT-PCR experiments, Trizol reagent (Sangon Biotech, China) was used to extract the total RNA, and the PrimeScript™ RT Reagent Kit with gDNA Eraser (RR047A, Takara, Japan) were used to reverse transcribe the total RNA into cDNA. qRT-PCR was performed using TB Green™ Premix Ex Taq™ (RR420A, Takara, Japan) and carried out in a CFX96 Touch Real-Time PCR Detection System (Bio-Rad, USA). The following conditions were used for amplification: 95 °C for 30 s, followed by 40 cycles of 95 °C for 5 s and 60 °C for 30 s. The sequences of the primers used for qRT-PCR are shown in Table S11. The amplicons were subjected to melt curve analysis to check the specificity of the amplified products. The relative expression level of each gene was calculated using the 2^−(ΔΔCt)^ method [69], using the gene encoding glyceraldehyde-3-phosphate dehydrogenase (*gapdh*) as a reference gene, and the gene repeated on each board.

## Supplementary Information

**Additional file 1.**

## Data Availability

The data was presented in the manuscript and the supporting materials. The raw reads data was submitted to the Short Read Archive (SRA) under the accession number SRR12516455, SRR12516456, SRR12516457 and SRR12516458 and BioProject accession number PRJNA659277.

## Abbreviations

3 M: 3-Month–old males
15 M: 15-Month–old males
3F: 3-Month-old females
15F: 15-Month–old females
COG: Clusters of orthologous groups
DEGs: Differentially expressed genes
*cyp17α*: Cytochrome P450 17-hydroxylase/17, 20 lyase
*cyp19α*: Cytochrome P450 aromatase
E2: Estradiol
FC: Fold-change
FDR: False-discovery rate
*fsh*: Follicle-stimulating hormone
*fshr*: Follicle-stimulating hormone receptor
*gapdh*: Glyceraldehyde-3-phosphate dehydrogenase
GO: Gene ontology
*hsd*: Hydroxysteroid dehydrogenase
*hsp*: Heat shock protein
KEGG: Kyoto encyclopedia of genes and genomes
KOG: EuKaryotic orthologous groups
*lh*: Luteinizing hormone
*lhcgr*: Luteinizing hormone/choriogonadotropin receptor
PGCs: Primordial germ cells
qRT-PCR: Quantitative reverse transcriptase PCR
*sox*: SRY-related HMG-box
T: Testosterone
