# Supplementary material for: Analyzing the gonadal transcriptome of the frog Hoplobatrachus rugulosus to identify genes involved in sex development
Source: BMC Genomics. 2021 Jul 19;22:552. doi: 10.1186/s12864-021-07879-6 (PMC8290591; doi:10.1186/s12864-021-07879-6)
Supplement: Supplementary file 1 — Additional file 1. [file 12864_2021_7879_MOESM1_ESM.zip › Supplemental Files.doc]

**Analyzing the gonadal transcriptome of the frog *Hoplobatrachus rugulosus* to identify genes involved in sex development**

**Yun Tang, Jing-Yi Chen, Guo-Hua Ding, Zhi-Hua Lin**

**Figure S1** **The Venn diagram of annotations in four major databases (KEGG, KOG, Nr and Swissprot).**

**Figure** **S2 Histogram of species distribution (showing only the top 10 species).**

**Figure S3 Functional classification diagram of KOG in gonads from both sexes of *Hoplobatrachus rugulosus* at two ages.**

**Figure S4 The histogram (a), scatter diagrams (b) and volcano plots (c) analysis of the DEGs in gonads from both sexes of *Hoplobatrachus rugulosus* at two ages.** 3F: 3-month-old female, 15F: 15-month-old female, 3M: 3-month-old male, 15M: 15-month-old male. The number of genes are shown on each bar in figure a.

**Table** **S1 Summary of data filtering** **for each sample.**

| Sample | Before filter reads number | After filter reads number (%) | GC % |
| --- | --- | --- | --- |
| 3-month-old females | 72,739,524 | 69,155,150 (95.07%) | 47.92 |
| 3-month-old males | 94,705,856 | 91,416,254 (96.53%) | 45.92 |
| 15-month-old females | 89,415,312 | 81,576,146 (91.23%) | 46.19 |
| 15-month-old males | 88,413,640 | 80,734,092 (91.31%) | 45.92 |

**Table S2 Statistical table of filtered base information.**

| Sample | After filter data (bp) | Q20 (%) | Q30 (%) |
| --- | --- | --- | --- |
| 3-month-old female | 9,179,237,556 | 8,552,773,173  (93.18%) | 7,769,110,364  (84.64%) |
| 3-month-old male | 12,883,037,993 | 12,155,948,450  (94.36%) | 11,145,536,336  (86.51%) |
| 15-month-old female | 11,418,933,844 | 10,487,245,933  (91.84%) | 9,399,069,722  (82.31%) |
| 15-month-old male | 11,277,919,322 | 10,363,255,717  (91.89%) | 9,302,594,715  (82.48%) |

**Table S3 Statistical table of assembly results.**

| Genes number | GC % | N50 | Max length | Min length | Average length | Total assembled bases |
| --- | --- | --- | --- | --- | --- | --- |
| 49,149 | 43.76 | 2455 | 24,170 | 201 | 1,593 | 78,334,903 |

**Table S4 The number of expressed genes for each sample.**

| Sample | Genes number | Ratio |
| --- | --- | --- |
| 3-month-old females | 42,502 | 86.48% |
| 3-month-old males | 48,910 | 99.51% |
| 15-month-old females | 45,289 | 92.15% |
| 15-month-old males | 48,588 | 98.86% |

**Table S5 Annotated statistics for the four** **major databases.**

| Total unigenes | Nr | Swissprot | KOG | KEGG | Annotation genes | Without annotation gene |
| --- | --- | --- | --- | --- | --- | --- |
| 49,149 | 20,925 | 15,392 | 11,982 | 12,973 | 20,942 | 28,207 |

**Table S10 Snout-vent length and body mass of each selected sample.**

| Species voucher | Sex | Age | Snout-vent length (mm) | Body mass  (g) |
| --- | --- | --- | --- | --- |
| LSU2015001 | Female | 15 months | 116.08 | 196.81 |
| LSU2015002 | Female | 15 months | 136.10 | 247.56 |
| LSU2015003 | Female | 15 months | 118.29 | 232.62 |
| LSU2015004 | Male | 15 months | 101.21 | 135.90 |
| LSU2015005 | Male | 15 months | 106.29 | 109.42 |
| LSU2015006 | Male | 15 months | 111.18 | 134.79 |
| LSU2015007 | Female | 3 months | 111.64 | 102.00 |
| LSU2015008 | Female | 3 months | 104.52 | 102.59 |
| LSU2015009 | Female | 3 months | 101.66 | 96.21 |
| LSU2015010 | Male | 3 months | 86.47 | 56.88 |
| LSU2015011 | Male | 3 months | 89.22 | 70.77 |
| LSU2015012 | Male | 3 months | 91.57 | 72.17 |

**Table S11 List of primers for qRT-PCR.**

| Target gene | Gene Name | Genbank accession number | Forward primer  (5’ to 3’) | Reverse primer  (5’ to 3’) | TM  (ºC) | Size  (bp) |
| --- | --- | --- | --- | --- | --- | --- |
| Unigene0042268 | *gapdh* | MN482674 | TTCTCCAGGCGGCAGGTCAG | CAAGGCTGTCGGCAAGGTCATC | 60 | 111 |
| Unigene0002432 | *soat1* | MT922038 | ATGCCTATCGGGACTTCCTCTGG | GGACCACAGCGGACACAGTAAAC | 60 | 87 |
| Unigene0016363 | *cyp3α* | MT922039 | GGCAGGATATGAGACAACCAGCAC | GGGAGGAGCCTTGTTGGGTAAATG | 60 | 124 |
| Unigene0005558 | *cyp17α* | MT922040 | CGCTGTGTATGTTCGGTGAAGGG | TGTCTGGAAGGATGTGAGGGTCTG | 60 | 95 |
| Unigene0004059 | *cyp19α* | MT922041 | CGCAAGGCTCTGGAGGATGAC | CAGGCTGAACTCATTTGGCTTTGG | 60 | 123 |
| Unigene0039250 | *hsd3β* | MT922042 | GCACAGAGGACACGGAGTATGAC | CGCCATCTTGGAGCACCTGAC | 60 | 111 |
| Unigene0004175 | *hsd11β1* | MT922043 | TGTGGGAGGATCTGGGTCATTTGG | ACGACAATGCTGCCTTGGGATTTC | 60 | 144 |
| Unigene0034720 | *hsd17β12* | MT922044 | AGCCGTGCCTGCTGGAGAG | GCCTTCCCTATGCCATCTGTAGC | 60 | 190 |
| Unigene0010920 | *sox2* | MT922046 | CGTTGTCACCTCTTCCTCTCACTC | GGCACTTGGCTCTGGCACTTC | 60 | 106 |
| Unigene0039185 | *sox9* | MT922047 | GAACGGGCAGGCTGAACAAGAG | TGGAGGCAGAGTGTGGAGAGTC | 60 | 101 |
| Unigene0010590 | *sox30* | MT922048 | TCCATCAACAGCAGCACTTCCTG | TGATTGACTTGAGGGTTGGCTTGG | 60 | 138 |
| Unigene0015026 | *hsp70* | MT922049 | GCATGGCAAGGTGGAGATCATCG | TGGCATCAAAGACCGTGTTCTGTG | 60 | 146 |
